# Measures for intersectional mental healthcare: results from a participatory interview and focus group study with psychosocial counsellors, people with experience of using mental health services and providers

**DOI:** 10.1192/bjo.2026.12026

**Published:** 2026-08-10

**Authors:** Mirjam Faissner, Patiani Batchati, Neneh Rosalía Quadflieg, Alva Träbert, Eike Leidgens, Georg Juckel, Jakov Gather, Amma Yeboah

**Affiliations:** Institute for History of Medicine and Science Studies, University of Lübeck, Lübeck, Germany; Department of Psychiatry, Psychotherapy and Preventive Medicine, LWL University Hospital Bochum, Ruhr University Bochum, Bochum, Germany; Institute for the History of Medicine and Ethics in Medicine, Charité – University Medicine Berlin, Berlin, Germany; Racism-critical Psychotherapy Association e.V., Berlin, Germany; German Association of Psychosocial Centers for Survivors of Torture, War, and Forced Displacement, Berlin, Germany; Medical Refugee Aid Bochum e.V., Bochum, Germany; Institute for Medical Ethics and History of Medicine, Ruhr University Bochum, Bochum, Germany; Independent Scholar, Cologne, Germany

**Keywords:** Racism, marginalised groups, LGBTQ, structural discrimination, healthcare delivery

## Abstract

**Background:**

Discriminatory practices and barriers to care are increasingly recognised as a significant challenge for mental healthcare institutions. Yet, little research has considered what measures people with experience of using mental health services and mental healthcare service providers recommend to implement anti-discrimination within mental healthcare.

**Aims:**

This study aims to explore the perspectives and recommendations of marginalised people with experience of using mental health services, mental healthcare service providers and psychosocial counsellors, to inform more equitable mental healthcare.

**Method:**

Between May 2022 and June 2023, we conducted 17 semi-structured qualitative interviews and 2 focus groups with psychosocial counsellors, people with experience of using mental health services and mental healthcare service providers. Data analysis followed structuring qualitative content analysis, combined with participatory methods.

**Results:**

Study participants recommended interpersonal, organisational and structural measures to address mental healthcare inequities. On an interpersonal level, they emphasised providers’ behaviour and attitudes toward discrimination. Organisationally, they called for a comprehensive intersectional care framework, fostering inclusive therapy environments, increasing staff diversity and implementing continuous anti-discrimination training. Structurally, they advocated for discrimination-critical therapy materials, revised curricula and removing barriers to medical education and healthcare.

**Conclusions:**

The study yielded suggestions for anti-discriminatory, critical consciousness-based and inclusive practices on the interpersonal, organisational and structural levels of mental healthcare. It offers important insights for research devoted to implementing and evaluating such measures.

The Code of Ethics of the European Psychiatric Association (EPA)^1^ stresses the role of mental healthcare service providers in addressing discrimination, and highlights this as an important aspect of ethical professional conduct: psychiatrists should never participate in or support discriminatory practices ‘on the basis of age, race, ethnicity, nationality, religion, sex, gender, sexual orientation, social standing, criminal background, disability, disease, or political affiliations’.^1^ At the same time, a growing body of research highlights that structural forms of discrimination, such as racism, impact mental healthcare.^2–4^ Research shows that marginalised people with experience of using mental health services, such as members of racialised groups,^5,2,6^ migrants and refugees,^7^ LGBTQ individuals,^8^ sex workers^9^ and people with disabilities,^10^ face barriers in accessing mental healthcare, whereas discriminatory practices within the system impact the quality of the care received.^11^ In addition, systematic reviews document that Black men are more likely to receive a diagnosis of psychosis^12^ and are more strongly affected by compulsory admission and coercive measures.^a^ ^,13–16^ Empirical research highlights the role of Othering through exclusion, access barriers, stereotyping, lack of time and resources, lack of interpretation services, and insufficient competencies on structural discrimination by healthcare service providers, with painful and potentially traumatic effects for those affected.^17–19^

Although the EPA task force on racial discrimination and mental health stresses the important role of healthcare service providers in mitigating discriminatory practices,^2^ clinicians’ ethical commitments may conflict with structurally embedded systems of discrimination that shape care delivery. Although research increasingly examines the forms, mechanisms and effects of structural discrimination in mental healthcare, little is known about how different stakeholder groups assess potential countermeasures in clinical care, and to the best of our knowledge, no such study has yet been conducted in the German context. This qualitative study addresses this gap by examining the following research questions: which measures to reduce discriminatory practices in mental healthcare are recommended by different stakeholder groups, including mental healthcare service providers and marginalised people with experience of using mental health services? How are these measures evaluated in terms of appropriateness and feasibility?

## Method

### Study design, methodological rationale and framework

We conducted a qualitative interview and focus group study to explore discriminatory practices in the German mental healthcare system and to identify potential measures to address them. The study was carried out as a collaboration between the Department of Psychiatry, Psychotherapy and Preventive Medicine at Ruhr University Bochum, the Institute for Medical Ethics and History of Medicine at Ruhr University Bochum, and two psychosocial centres for marginalised social groups in Bochum, Germany: Rosa Strippe e.V. and Medical Refugee Aid Bochum (Medizinische Flüchtlingshilfe Bochum e.V.).

The study comprised two phases. In phase 1, we conducted semi-structured, problem-centred interviews^20^ to explore discriminatory practices in mental healthcare from different stakeholder perspectives. The data were analysed using a constructivist grounded theory approach.^21^ These findings have been published elsewhere.^22^ In phase 2, all participants from phase 1 were invited to take part in focus groups, where they were presented with the phase 1 results and asked to collaboratively develop and discuss measures to address discrimination in mental healthcare. The focus groups as well as the interview data were analysed using structuring content analysis informed by Kuckartz^23^ to identify measures for equitable mental healthcare. The results of this analysis are presented in this paper.

A qualitative approach was chosen as it is particularly suited to examining underresearched phenomena in a context-sensitive manner, and to generating new insights grounded in the experiences of actors differently positioned within the field.^23,24^ Furthermore, a participatory approach was realised via a study team that involved non-academic researchers from the local non-governmental organisations Rosa Strippe e.V. and Medical Refugee Aid Bochum to jointly coordinate the study, member checking conversations and participatory data analysis sessions with study participants. The participatory approach was grounded in epistemic and normative considerations: it aimed to deepen understanding of the lived, intersectional realities of discrimination and to involve structurally disadvantaged groups in research that directly concerns them for reasons of structural and epistemic justice.^25–27^

This study adopts an intersectional approach to address structural discrimination, focusing on the intersections of racism, discrimination based on gender and sexual diversity, and sanism in mental healthcare. We understand discrimination as systematic disadvantage or harm that accrue for individuals based on salient social group membership.^28^ It is structural if it is embedded in the very ‘structures’ of a society, including common forms of social interaction, institutional processes, legal frameworks, social norms and socially shared knowledge, leading to the marginalisation of these groups.^29^ We use the term ‘discriminatory practices’ to refer to patterned forms of social interaction based on formal and informal rules that reproduce discrimination, regardless of intent.

Intersectionality has proven a powerful approach for analysing such structural dynamics. Stemming from a long tradition within grassroots organising and Black feminist activism, legal scholar Crenshaw^30^ coined the term ‘intersectionality’ to highlight how different systems of structural discrimination, such as sexism and racism, unfold in simultaneity, leading to specific social positions of disadvantage or privilege within complex societal systems. In this study, intersectionality informed data collection, analysis and overall research practice.^24^

### Participant sampling

We included three participant groups: (a) adult people with experience of using mental health services with reported experiences of discrimination within mental healthcare and a self-reported mental health diagnosis; (b) mental healthcare service providers and (c) psychosocial counsellors with counselling expertise in people with experience of using mental health services who have been affected by discrimination.^b^ In line with an intersectional approach, experience of discrimination was defined broadly as self-reported instances of disadvantage or harm within mental healthcare services based on one or more identity markers. In phase 1, we employed a theoretical sampling strategy to capture diverse perspectives across intersections of treatment experiences, professional roles, gender, racialisation, sexual orientation, ability and socioeconomic status. This approach aimed to ensure representation of varied experiences relevant to the study objectives. In phase 2, all phase 1 participants were invited to participate in the focus groups.

### Recruitment

We recruited participants via invitation emails sent to psychosocial community centres and the psychiatry network of the Regional Association of Westphalia-Lippe (Psychiatrie Verbund des Landschaftsverbands Westfalen-Lippe), a large mental healthcare service provider in Western Germany. Additionally, flyers were distributed via the collaborating organisations. People interested in study participation contacted the first author via phone or email and received verbal information on study participation. Eligibility criteria were checked during this preliminary telephone call, where potential participants from the group of people with experience of using mental health services were invited to briefly describe the kind of discrimination encountered in mental healthcare. All potential participants met the eligibility criteria.

All participants received written information about the study and gave their written informed consent to participate in the study. The authors assert that all procedures contributing to this work comply with the ethical standards of the relevant national and institutional committees on human experimentation and with the Helsinki Declaration of 1975, as revised in 2013. All procedures involving human patients were approved by the Research Ethics Committee of the Medical Faculty of the Ruhr University Bochum (registration number 22-7504).

### Sample characteristics

Seventeen participants, comprising three psychosocial counsellors, seven mental healthcare service providers and seven people with experience of using mental health services were included (see Table 1). The mental healthcare service providers included three psychiatric residents and one psychiatric consultant, one psychological therapist and two psychiatric nurses, with a mean professional experience of 24 years (s.d. = 15). Three of the mental healthcare service providers and all of the psychosocial counsellors also reported experiences of discrimination based on one or more identity markers. The people with experience of using mental health services reported a broad range of psychiatric diagnoses according to the ICD-10 classification, with depressive disorder being the most prevalent. The time of initial diagnosis among people with experience of using mental health services ranged from 2015 to 2022, and the number of in-patient psychiatric treatments varied between one and four.

**Table 1:** Sociodemographic characteristics of the study participants: people with experience of using mental health services who have been affected by discrimination (*n* = 7), mental healthcare service providers (*n* = 7) and psychosocial counsellors (*n* = 3) Table 1 long description.

Variable (self-description)	*n*	%
Age, mean in years (s.d.)	41 (10)	
Gender identity		
Woman	4	24
Man	9	53
Trans woman	1	6
Trans	3	18
Non-binary	3	18
Rejection of gender categorisation	1	6
Sexual orientation		
Homosexual	3	18
Bisexual	1	6
Asexual and queer	1	6
Pansexual	2	12
Queer	4	24
Heterosexual	3	18
Androphilic	1	6
No information	4	24
Racialisation		
Person of colour	3	18
Arab	2	12
Black	1	6
Latinx	1	6
*white*	12	71
Russian-German	1	
Disability		
No	12	70
Yes	4	24
Perceptible to others	0	0
Imperceptible to others	4	100
No information/would not like to comment	1	6

The sum of the percentages does not add up to 100% because of multiple answers.

### Data collection

Data collection took place between May 2022 and June 2023. In study phase 1, we conducted 17 semi-structured interviews.^20,22^ In study phase 2, we conducted two facilitated focus group discussions. In addition, all participants filled out a sociodemographic questionnaire, where they could choose categories about their social identity from a list, add their own self-identification and choose as many categories as they wished. The suggested categories reflected different prevalent forms of racism in Germany, including anti-Muslim and anti-Slavic racism.

#### Qualitative interviews

We conducted problem-centred interviews as proposed by Witzel.^20^ This method allowed exploring the specific topic of intersectional experiences of discrimination in the context of mental healthcare. Conducting individual interviews provided a confidential setting that enabled participants to share potentially traumatic or distressing experiences in a protected environment.

The study team developed separate interview guides for each of the three stakeholder groups based on a review of the existing literature. We first conducted the interviews with psychosocial counsellors specialised in anti-discrimination work and presented them with the draft interview guides for the other stakeholder groups. The guides were subsequently revised based on their feedback. The final interview guides included questions on experiences of discrimination in mental healthcare, their perceived effects and suggestions for improving mental healthcare services.^c^ We conducted the interviews in a tandem of two researchers. One interview was professionally interpreted from Arabic to German. The interviews lasted 59 min on average (s.d. = 14 min).

#### Focus groups

All study participants from phase 1 were invited to participate in a 90 min focus group to discuss measures for intersectional mental healthcare. Based on the preliminary results from phase 1, we conducted one focus group centring on racism (*n* = 5), and one centring on gender and sexual diversity (*n* = 5) in mental healthcare. Focus groups were chosen because they are particularly suited to develop context-sensitive understanding of social experiences based on the interaction of multiple perspectives. In addition, they enable the identification of shared norms and values, as well as the joint generation of tailored solutions to collaboratively identified challenges.^31^ The guide used for both focus groups was developed by the research team based on phase I results, and can be consulted in Supplementary Appendix A.

Participants were first presented with the main results of phase 1 that included a description of identified discriminatory practices on the interpersonal, institutional and structural level. They were asked to reflect on these findings and indicate whether they found them plausible and consistent with their own experiences. Subsequently, participants were invited to suggest and discuss potential measures to address discrimination at the individual, institutional and structural levels, and to evaluate these measures in terms of their perceived appropriateness and feasibility.

The interviews and focus groups were audio-recorded, transcribed verbatim by an external transcription service, and pseudonymised. All participants were offered to receive their interview transcript, and were invited to participate in a non-structured member-checking conversation with the first author, to discuss the transcript and preliminary interpretation. People with experience of using mental health services were offered a compensation of €25 for the interview, €15 for a member-checking conversation and €20 for the participation in a focus group. M.F. and P.B. translated quotes from German to English, and A.T., a bilingual speaker, proofread the translation.

### Data analysis

We conducted structuring qualitative content analysis informed by Kuckartz,^23^ combining deductive and inductive category development. MAXQDA Standard Portable (version 22 for Windows; VERBI Software GmbH, Berlin, Germany; available from: https://www.maxqda.com/de/) was used for data analysis.

M.F. and P.B. first familiarised themselves with the focus group transcripts. Based on the findings from phase 1, a review of the literature and the research questions, they developed and defined the main categories. Both researchers independently coded the two focus groups using these main categories, and compiled the coded text segments for each category. Within the main categories, subcategories were then developed inductively from the focus group data. The category system was subsequently revised and hierarchically structured; all categories were defined and supplemented with anchor quotations. M.F. and P.B. met regularly to discuss and refine the coding framework. Coding discrepancies were discussed until consensus was reached, and the category system was presented and discussed with all co-authors. The refined coding system was then applied to the focus group material as well as relevant sections of the phase 1 interviews addressing suggestions for anti-discrimination measures in mental healthcare. Finally, M.F., P.B. and A.Y., reviewed all interview and focus group material and selected illustrative quotations representing the respective categories.

### Researcher reflexivity

The study team consisted of one academic researcher, one medical doctor, one master student and two psychosocial counsellors from the collaborating local community centres. The researchers had academic backgrounds in medicine, psychology, history, sociology and philosophy. They were supervised by a psychiatric specialist and psychotherapist, and two psychiatric consultants. The principal investigator (M.F.) has supervised and performed a range of qualitative research projects,^11,32^ and all researchers involved in the data analysis were trained by the Methods Centre of Ruhr-University Bochum in qualitative data analysis. The authors identify as *white* (*n* = 5), Black (*n* = 2), Latinx (*n* = 1), male (*n* = 3), female (*n* = 4) and queer (*n* = 4).^d^ Regular reflection meetings helped the study team identify personal biases and implicit background assumptions on the study topic. They were also helpful in repeatedly raising awareness of the researchers’ own social positioning within academic and societal power structures and in considering how this might influence the study results. More detailed information on researcher reflexivity can be found in Supplementary Appendix B.

## Results

We identified 14 categories of measures for intersectional mental healthcare on the interpersonal, organisational and structural levels, which are highlighted in bold throughout the text. Tables 2 to 4 provide an overview of these categories and their definitions, with exemplary quotes illustrating key categories in the text.

While participants’ accounts of discriminatory practices referred to specific, intersecting identity markers, including gender and sexual identity, racism, religion, sanism and neurodiversity, the proposed measures were formulated at a more general level and conceived as suitable to addressing multiple, intersecting forms of discrimination. To enhance clarity of the results, Tables 2 to 4 also include illustrative examples of discriminatory practices in mental healthcare identified in phase 1 (quoted from Quadflieg et al^22^), with sanism, racism and discrimination based on gender and sexual diversity emerging as the most frequently reported forms.

**Table 2:** Description of interpersonal-level measures for intersectional healthcare (phase 2) and their alignment with discriminatory practices identified in phase 1 retrieved from Quadflieg et al^22^ Table 2 long description.

Proposed interpersonal-level measures		Discriminatory practices
Measure	Description	
**Respectful attitude**	Treating SUs affected by racism as an individual person rather than as a representative of a group or culture Taking reported experiences of discrimination seriously	**Stereotyping**: Service provider stereotypes (‘Yes, I know, you Turks […]’) **Invalidation of trans and non-binary identities**: Misgendering and deadnaming; refusal to address non-binary user beyond ‘Mrs’ **Invalidation of experiences of racism**: Therapist questioned racism reports (‘Do you really think that’s racist? Maybe the person was in a bad mood?’)
**Signalling support**	Proactively signalling support to marginalised SUs to make them feel safe enough to disclose their concerns, e.g. through the use of gender-inclusive language	**Negative consequences of silencing on well-being**: Provider (*white*, cis man) linked having to ‘keep quiet about what actually moves me’ to mistrust, inability to ‘open up emotionally’ and ‘suicidal crises’
**Humility regarding one’s expertise and authority**	Openly admitting one’s fallibility and recognising the limits of one’s own competencies as a competence Actively seeking feedback from SUs Considering SUs as experts and valuing their experiential knowledge	**Failure to address SU needs because of gender bias**: SU (*white*, trans and non-binary) was transferred during an acute suicidal crisis without assessment; in the emergency room, a doctor offered ‘treatment’ for their gender identity instead of addressing suicidality **Gender-normative bias in care**: SU (*white*, trans man) reported that his wish to carry a child and breastfeed was invalidated; his therapist expected ‘authentic’ transness to align with stereotypical masculinity, questioning his trans identity

SU, people who used mental health services.

**Table 3:** Description of organisational-level measures for intersectional healthcare (phase 2) and their alignment with discriminatory practices identified in phase 1, retrieved from Quadflieg et al^22^ Table 3 long description.

Proposed organisational-level measures		Discriminatory practices
Level	Measure	
**Intersectional therapy environment**	Providing information materials in multiple languages Offering interpretation services Considering religious needs of SUs, e.g. prayer rooms, time for prayer and catering to dietary requirements Introducing appointment scheduling systems that reduce the risk of discrimination, e.g. via online systems	**Structural language barriers and denial of care**: an SU (asylum seeker, racialised, cis man) received no treatment for days and no translated information (e.g. food access). Admission was often denied because of limited German language; SUs were told to bring relatives as interpreters (confidentiality concerns), and no extra time was provided for complex communication **Cis-normative and exclusionary infrastructures**: Room assignments followed assigned sex at birth; an SU (*white*, trans woman) reported that being forced to sleep in a men’s room hindered recovery. IT systems could not record correct names/pronouns, leading to repeated misgendering **Hostile and minimising institutional climate**: Homophobic/transphobic materials were displayed; interpreters lacked LGBTQIA+ competence; exclusion was justified as affecting ‘only a small number’ or being ‘too time consuming’
**Group-specific therapy offers for marginalised populations**	Implementing group therapy or peer-led round tables for SUs affected by structural discrimination, e.g. with a focus on racism or gender diversity, and offering a diverse range of therapeutic offers, e.g. art therapy	**No space for marginalised experiences in therapy**: SU (Person of colour, lesbian, atheist) reported that therapy was less effective because she could not discuss her experiences of racism, or refugee background in group or individual sessions; her identity and experiences did not ‘fit’ the providers’ expectations
**Public commitment against discrimination**	Active involvement of leadership in anti-discrimination Investing resources in building a functioning infrastructure for anti-discrimination Implementing transparent feedback mechanisms and procedures for cases of discrimination	**Lack of infrastructure to address discrimination**: Racialised, non-binary SU felt unable to name ‘discrimination’ or ‘racism’ by a senior physician, fearing negative consequences for ongoing therapy
**Diversity among staff**	Adopting diversity-oriented hiring practices, creating a welcoming work environment for professionals with a socially marginalised identity	**Insufficient understanding in *white* German work environment**: One service provider (Latinx, Person of colour, cis man) reported that co-workers minimised his report of racist discrimination: *’White* Germans somehow don’t take racism seriously enough… [they say:] “there is no racism here in Germany and it’s just a small thing”’
**Supervision and intervision**	Regularly discussing sensitive topics (e.g. questions of whether something is racist or trans-hostile) within the team, supervisors or with external experts	**Inadequate critical reflection and sensitivity among service providers**: SUs reported staff becoming defensive when confronted about discriminatory practices; gendered and racialised mental illness stereotypes were used to invalidate criticism (‘overly sensitive’)
**Mandatory, continuous and practical educational formats**	Establishing a system of regular mandatory workshops to ensure that healthcare workers remain updated (‘lifelong learning’) Focusing on practical, skill-oriented learning	**Limited provider skills on discrimination**: Some service providers acknowledged lacking skills to address racism or its mental health impact and recognised that their judgements were sometimes shaped by negative stereotypes
**Standard operating procedures (SOPs)**	Establishing SOPs and revisiting them in training sessions to ensure ethical uniform practices where healthcare professionals may feel uncertain (e.g. handling pronoun use and room assignments for trans SUs, addressing experiences of racism in psychotherapy)	**Absence of guidelines on gender diversity**: One service provider (*white*, cis man) described trans users facing access barriers. Discriminatory room assignments were linked to the absence of institutional guidelines on gender diversity

SU, people who used mental health services.

**Table 4:** Description of structural-level measures for intersectional healthcare (phase 2) and their alignment with discriminatory practices identified in phase 1, retrieved from Quadflieg et al^22^ Table 4 long description.

Proposed structural-level measures		Discriminatory practices
Measure	Description	
**Inclusive and discrimination-critical therapy materials**	Developing and implementing assessment tools for discrimination that include relevant questions about factors such as everyday discrimination, personal or family history of migration, religion and gender identity, and that allow mental healthcare service providers to directly and systematically address the topic of discrimination	**Limited awareness and availability of discrimination-sensitive tools**: One provider (Black, cis woman) explained that many psychotherapists are unaware that existing tools (Z-Codes in the ICD) allow recording SUs’ experiences of discrimination and their impact on mental health. She also highlighted the absence of German-language resources and manuals addressing racism in psychotherapy.
**Discrimination-critical therapy concepts**	Developing therapy concepts that focus on the impact of structural discrimination on mental health Introducing diverse recovery assistants or peer workers in psychiatric settings to reduce discrimination and stereotyping related to mental illness among staff members and to provide SUs with support from someone with similar experiences	**Absence of racism-sensitive therapy concepts**: One provider (Black, cis woman) noted that ‘racism-sensitive therapy … just doesn’t exist’ in Germany. **Invalidation by service providers (sanism, sexism, racism)**: SUs reported staff using gendered and racialised mental illness stereotypes to dismiss criticism, e.g. calling SUs ‘overly sensitive’.
**Address barriers to medical school admissions**	Critically reassessing the exclusive selection criteria to enter medical school, e.g. the grade-based criteria in Germany	
**Address barriers to healthcare access**	For governments, authorities and associations of statutory health insurance physicians: acknowledging the complexity of barriers to healthcare and making a significant effort in reducing these now, both in terms of energy and funding	**Mental healthcare institutions as exclusive spaces**: One service provider (Black, cis woman) stated that ‘the most important discrimination… is that people don’t even arrive at all… certain people arrive in psychiatric wards and others just don’t’.

SU, people who used mental health services.

In quotations in the Results section, ‘SU’ denotes ‘people with experience of using mental health services’, ‘MHP’ denotes mental healthcare service provider, ‘PC’ denotes psychosocial counsellor, ‘FG_R’ denotes the focus group on racism and ‘FG_GDS’ denotes the focus group on gender and sexual diversity. Identity markers relevant to each quotation are indicated in parentheses.

### Interpersonal level

People with experience of using mental health services requested a **respectful attitude** from mental healthcare service providers and stressed that all people should be treated as individuals rather than as representatives of a group or culture. Various racialised and trans people with experience of using mental health services and providers highlighted that reported experiences of discrimination need to be taken seriously without being scrutinised by staff. Participants suggested that healthcare service providers need to proactively **signal their support** to marginalised people to make them feel safe enough to disclose their concerns. One person with experience of using mental health services shared that he has had ‘shit experiences’ and would therefore ‘never bring up the trans topic in therapy these days’, unless they ‘hear something […] queer-friendly’ from their practitioner (SU2, *white*, trans man).

Participants also stressed that healthcare service providers must acknowledge their responsibility in power dynamics and emphasised the importance of **humility** regarding one’s expertise and authority as a mental healthcare service provider. One psychotherapist stressed the importance of openly admitting one’s fallibility to ‘attempt to break down hierarchical structures [between SUs and providers]’ (MHP6, Black, cis woman). Healthcare service providers should understand recognising ‘the limits of one’s own competencies as a competence’ (FG_GSD). Focus group participants discussed this idea under the term ‘competency at lacking competency’ (*‘Kompetenzlosigkeitskompetenz*’), as described by the critical race scholar and educator Paul Mecheril (FG_GSD). One psychotherapist highlighted the importance of actively seeking feedback, telling all her clients upfront, ‘You have to give me feedback […] I can’t work without it, because I’m only human. I make […] many mistakes’ (MHP6, Black, cis woman). By contrast, healthcare service providers should consider patients as experts and value their experiential knowledge, as noted by one person with experience of using mental health services: ‘I expect them to listen to me and to believe that I, as a patient, can also bring something to the table’ (SU3, Arab, non-binary).

### Organisational level

Several participants stressed the necessity to create an **intersectional therapy environment** that allows people with diverse social identities to feel at ease. Psychosocial counsellors stressed that this involves providing information materials in multiple languages to ensure accessibility for all people, as well as providing interpretation services. Participants also mentioned that the religious needs of various groups should be considered. They suggested providing prayer rooms, allowing time in the schedule for prayer, and catering to dietary requirements in hospital meal planning. A doctor recommended non-discriminatory appointment scheduling systems to reduce barriers to healthcare, e.g. via online systems (MHP1, *white*, cis man).

Participants also suggested that psychiatric hospitals and clinics should provide **group-specific therapy offers** tailored to the mental healthcare needs of marginalised populations. For instance, hospitals could implement group therapy or peer-led round tables for racialised or trans and non-binary people. Racialised people with experience of using mental health services stressed that art therapy would be particularly helpful for marginalised service users to help express experiences that are difficult to put into words.

A **commitment** to working against discrimination could become evident through leadership’s active involvement. Participants stressed that in addition to publishing anti-discrimination commitment statements, institutions should invest resources in building a functioning infrastructure for anti-discrimination. This involves implementing transparent feedback mechanisms and procedures for cases of discrimination.

Participants noted that **diversity among staff** was crucial. One doctor recommended recruitment policies at hospitals that actively encourage applications from marginalised groups and suggested that greater diversity could enhance trust among marginalised groups in their healthcare providers and positively impact treatment outcomes (MHP3, *white*, cis man). One person with experience of using mental health services confirmed: ‘If I notice something about a person that doesn’t fit the norm, I’m automatically more open’ (SU2, *white*, trans man). A psychotherapist shared her experience: ‘I had a person in the psychiatric ward who […] said: “I love working with you because you always remind me of my aunt”, and of course we need such a mild positive transference in therapy. She added that children reacted even more directly: ‘They’d say to me: “Man, we thought an old *white* woman was coming.” Nothing against *white* women […] But it just had an effect on the children’s minds’ (MHP6, Black, cis woman).

Participants also emphasised the importance of **supervision** and **intervision** in fostering a supportive and less discriminatory clinical environment. They addressed the value of supervision with external experts, who help to ‘guide and structure’ (FG_R), as well as the importance of discussing sensitive topics within the team. According to the participants, supervision could help navigate complex issues on which opinions may differ, such as questions of whether a practice is racist or hostile to trans people.

Several participants, including people with experience of using mental health services and mental healthcare service providers, advocated for **mandatory, continuous and practical educational formats** for staff. Focus group participants suggested a recurring mechanism of mandatory workshops to ensure that healthcare workers remain updated, stressing that ‘lifelong learning’ is necessary to avoid superficial understandings of complex topics like racism (FG_R). Without ongoing educational interventions, there is a risk that practitioners might develop a false sense of competence, mistakenly believing they are ‘multiculturally enlightened’, when, in fact, the field is constantly evolving and demands continuous engagement (FG_R). They also emphasised the importance of **practical, skill-oriented learning** rather than purely theoretical lectures.

Mental healthcare service providers also emphasised the need for **standard operating procedures** (SOPs) to ensure appropriate care for ‘certain [marginalised] SUs’ (MHP5, *white* and Russian-German, cis man). They expressed uncertainty about handling pronoun use and room assignments for trans people with experience of using mental health services. For instance, one doctor (MH3, *white*, cis man) was unsure whether gender diversity issues were addressed consistently throughout the clinic. Establishing SOPs and revisiting them in training sessions could help ensure ethical uniform practices.

### Structural level

Several mental healthcare service providers noted the absence of tools for assessing discrimination, highlighting, for instance, the importance of understanding the reasons behind delayed healthcare visits. One psychotherapist pointed out that existing therapy manuals are not appropriate for all mental healthcare help-seekers, as they often fail to include relevant questions about factors such as migration, religion or gender identity (MHP6, Black, cis woman). Although this psychotherapist made her own adjustments with her clients, she emphasised the necessity for **more inclusive and discrimination-critical therapy materials**. A person with experience of using mental health services stressed that this could enable mental healthcare service providers to directly and systematically address the topic of discrimination within therapy (SU4, *white*, trans woman).

Regarding the development of **discrimination-critical therapy concepts**, one interviewee shared their experiences with ‘recovery assistants’ or ‘peer workers’ in psychiatric settings. They found that including peer workers in the team helped reduce mental illness stereotypes and transformed related conversations. Furthermore, they emphasised that it was beneficial for people with experience of using mental health services to have someone with similar experiences available for support and suggested that this model could be expanded to address other forms of discrimination beyond sanism, such as racism or discrimination related to gender and sexual diversity (FG_GSD).

Participants identified a significant structural gap in the **design of training curricula**. They advocated for the inclusion of topics such as racism, ableism, classism, weight discrimination and gender and sexual diversity. One person with experience of using mental health services noted that meeting a doctor with knowledge and practical experience about gender diversity made a ‘huge difference’ for him (SU1, *white*, trans man). Several participants criticised that medical training often focuses on people with experience of using mental health services who fit certain societal norms, commonly referred to as the ‘YAVIS patient’: young, attractive, verbal, intelligent, and successful (MHP6, Black, cis woman). They expressed concern about the lack of attention given to individuals with cognitive disabilities and to those from the working class, and suggested that medical training should focus on the situation of marginalised people. One psychosocial counsellor stressed the importance of such knowledge since ‘we are all somehow socialised in a very heteronormative [racist, ableist …] society’ (PC1, *white*, non-binary).

Several participants expressed that lack of knowledge leads to service providers’ failure to recognise their own participation in discriminatory practices. One psychotherapist attributed the stability of this lack of acknowledgment to the widespread attitude: ‘If we can’t treat people, then they’re just not treatable’ (MHP6, Black, cis woman). One psychosocial counsellor recommended a non-profit’s self-learning platform as a useful tool for healthcare service providers to reflect on their role in structural discrimination (PC1, *white*, non-binary). Such platforms can offer an accessible way to learn about key topics related to discrimination.

Participants emphasised the need to address **barriers to medical school admissions**, criticising Germany’s grade-based selection system for excluding qualified, well-suited candidates. One interviewee advocated for a critical reassessment of these criteria, arguing that they hinder diversity and contribute to her being ‘a bit rare’ as a Black therapist in Germany (MHP6, Black, cis woman).

In addressing **barriers to healthcare access**, the same psychotherapist noted that certain people avoid seeking psychiatric care because of fears of not being able to discuss their concerns, past negative experiences or unmet needs for ‘queer-sensitive’, ‘cultural-sensitive’ or ‘religious-sensitive’ care. She emphasised that marginalised people with experience of using mental health services face a high ‘threshold to really make use of the healthcare system’. One psychosocial counsellor stressed the urgent need to reduce healthcare barriers, stating, ‘Health is a human right to which we [as a society] are currently not held accountable’. They acknowledged the complexity of addressing these barriers, emphasising the significant effort required: ‘So we simply have to take all access and treatment barriers really, really seriously, and that costs an incredible amount of energy and money’ (PC2, *white*, no gender category).

## Discussion

This qualitative interview and focus group study investigated ways to improve mental healthcare toward anti-discriminatory and intersectional practices based on the perspectives of marginalised mental health people with experience of using mental health services, psychosocial counsellors and mental healthcare service providers. The participants provided a rich description of anti-discriminatory, critical consciousness-based and inclusive practices on the interpersonal, organisational and structural levels of mental healthcare.

On the interpersonal level, people with experience of using mental health services requested respect, support and humility from healthcare service providers, suggesting that some healthcare service providers lack awareness of their participation in discriminatory practices. Studies on interventions to reduce interpersonal discriminatory behaviour in mental healthcare highlight approaches that enhance knowledge and awareness about mental health conditions and the impacts of stigma, targeting the reduction of biases.^33–35^ One approach to increase awareness of personal biases is by taking the Harvard Implicit Association Tests,^36^ which can function as a gateway to acknowledge and reflect on one’s own stereotypes. Another example is the brief online course *’*Implicit Bias in the Clinical and Learning Environment’ conducted at the University of Washington School of Medicine, which focuses on behavioural change.^37^ In a meta-analysis on interventions to reduce discrimination and stigma, programmes combining education, contact with stigmatised groups and targeting biases increase knowledge, awareness and intentions to change behaviour in healthcare professionals.^34^ Assessments for the effects of anti-discriminatory interventions show sustained positive change on short-term behaviours, although long-term behaviours have not been studied and therefore remain obscure.

Organisational-level measures proposed by study participants highlighted accessibility to care through the implementation of an intersectional therapy environment with specific therapeutic offers for marginalised groups and diversity among staff. To date, studies on intersectionality in interventional health research have mostly used intersectionality as an analytic tool to assess shortcomings, rather than as a practical approach to inform organisational redesign.^38^ Our phase 1 results highlighted that service providers shared high levels of frustration because of a lack of skills and tools to address organisational barriers to care, resulting in dissatisfying therapeutic relationships.^22^ In the experience of the last author as a supervisor, physicians describe feeling helpless when witnessing discrimination among colleagues and people with experience of using mental health services. Such experiences, as studies on moral distress show,^39^ may contribute to professional discontent and burnout, potentially leading to leaving clinical practice altogether. In this study, participants specifically advocated for diversity among healthcare professionals. However, although diversification of medical students and staff might strengthen people with experience of using mental health services’ trust to access healthcare resources, it should be noted that healthcare professionals also face varying dimensions of privilege and discrimination at educational and training institutions, as well as at the workplace.^40–42^ Healthcare organisations thus need resources and the drive to implement intersectionality-based principles and ethical codes of conduct, facilitate teambuilding and enforce empowerment and resilient strategies to create spaces that can contain all differential experiences of power, privilege, marginalisation and inclusion.

Structural-level interventions presented by study participants such as discrimination-critical assessment tools, as well as discrimination-critical therapy concepts show promising outcomes based on available materials. Regarding interventions targeting discriminatory practices from an intersectional perspective, Lazaridou and Fernando^6^ take a convincing stand for intersectional reflexivity as a practice of intersectionality-informed mental healthcare that focuses on the impact of structural violence on mental health, and recognises the societal and epistemic violence including everyday discriminatory practices against marginalised groups.^43^ The trained ability to identify how clinical symptoms, attitudes or diseases encountered in clinical practice can reflect the implications of wider structural factors, such as healthcare and food delivery systems, zoning policies, urban and rural infrastructures, medicalisation, and societal definitions of health and disease has been coined ‘structural competency’ by Metzl and Hansen.^44^ The relevant skill set that shapes structural competency have been standardised in an interdisciplinary pre-health curriculum called ‘Medicine, Health, and Society’ and assessed with the Structural Foundations of Health Survey tool.^45^ A study at Vanderbilt University shows that these skills develop through training rather than attitude change alone, and stresses the relevance of beginning training as early as possible.^46^ This confirms our study participants’ recommendation of on-going professional training in the sense of life-long learning. In sum, promising interventions on the structural level include cultural competency courses with a focus on critical consciousness and social inequity, checklists to assess the structural vulnerability of people with experience of using mental health services, as well as interdisciplinary partnership programmes with the legal and social sciences.^47,48^

When interpreting the results of this study, several limitations should be taken into account. First, although we initially aimed to capture a broad range of intersectional perspectives, experiences at the intersection of sanism, discrimination related to gender and sexual diversity, and racism were more frequently reported in phase 1. We attribute this to our institutional embeddedness and the networks of participating organisations, a focus that was further reinforced when the focus groups were centred on these experiences. In addition, participation required individuals to come forward to ensure voluntariness, which likely attracted those who felt sufficiently competent and safe to discuss their experiences and whose experiences were recognised as discrimination. As a result, other forms of discrimination, particularly less recognised ones such as bodyism, may be underrepresented. Given the many different forms of intersectional discrimination in mental healthcare, further research addressing additional forms of discrimination and their intersections is warranted.

Second, we did not systematically compare responses across different participant groups. Because focus groups included members from all three participant groups (people with experience of using mental health services, mental healthcare service providers and psychosocial counsellors), it was not possible to attribute specific results from the focus groups to particular types of participants. Future research on the implementation of measures to promote anti-discrimination in mental healthcare should explore these differences to assess their feasibility and acceptability across stakeholder groups.

Finally, participants were asked to discuss hypothetical anti-discrimination measures, meaning that their responses reflect assumptions rather than direct experiences with specific interventions. Although this approach provided insight into perceived feasibility and relevance, it provides limited conclusions regarding the practical implementation of such measures.

Our study provides important insights on strengthening anti-discrimination in mental healthcare from a German perspective. Participants’ recommendations and demands potentially require the painful realisation that we, as healthcare professionals and researchers, are not neutral with respect to structural discrimination in education, research and clinical practice. It also highlights the urgent need to move from theoretical and analytical frameworks to concrete interpersonal, organisational and structural interventions. Our results invite healthcare professionals to a space of personal reflexivity and critical consciousness, acknowledging that healthcare practices are a product of politics of structural violence and struggles for equity, and have never been, nor can ever be, neutral. The challenge for healthcare professionals on the interpersonal level therefore lies in our capacity to take personal responsibility for discriminatory practices, as well as accountability for reported experiences of discrimination. Organisational and structural interventions that shape our education, training and everyday practices are needed to guide, support and strengthen our ethical value of discrimination-free healthcare as a human right.

## Supporting information

10.1192/bjo.2026.12026.sm001 Faissner et al. supplementary material Faissner et al. supplementary material

## Data Availability

The data that support the findings of this study are available on request from the corresponding author, M.F. The data are not publicly available because they contain information that could compromise the privacy of research participants.

## Footnotes

a. In Germany, racial or ethnic categories are not routinely collected in official statistics, including registries on coercion. Instead, ‘migration background’, defined as a personal or parental history of migration from any country, is used as a proxy to assess discrimination, which does not adequately capture experiences of racism or racialisation.^16^ As a consequence, there is currently no data on the relationship between racialisation and rates of coercion in Germany.

b. In Germany, a psychosocial counsellor is a trained professional who provides structured counselling focused on how psychological factors interact with a person’s social situation and current life stressors.

c. The guides for the interviews can be downloaded in the Supplementary Material section of Quadflieg et al^22^.

d. We capitalise Black to emphasise its social construction and use as a political category. We lower case and italicise *white* to underscore that Whiteness is also socially constructed.

## Supplementary material

The supplementary material is available online at https://doi.org/10.1192/bjo.2026.12026

## Table 1 Long description

The table presents sociodemographic characteristics of seventeen participants, including three psychosocial counsellors, seven mental healthcare service providers, and seven people with experience of using mental health services. The mental healthcare service providers include three psychiatric residents, one psychiatric consultant, one psychological therapist, and two psychiatric nurses, with a mean professional experience of twenty-four years. The table also highlights experiences of discrimination among some healthcare service providers and counsellors. people with experience of using mental health services report a range of psychiatric diagnoses, with depressive disorder being the most prevalent. . Key data points include age distribution, gender identity, sexual orientation, racialization, and disability status among participants.

Navigate back to Table 1.

## Table 2 Long description

A table with three columns and multiple rows detailing interpersonal-level measures for intersectional healthcare. The columns are labeled Measure, Description, and Discriminatory practices. The Measure column lists categories such as Respectful attitude, Signalling support, and Humility regarding one’s expertise and authority. The Description column provides explanations for each measure, including treating people who used mental health services who have been affected by racism as individuals, proactively signaling support to marginalized people who used mental health services, and admitting one’s fallibility. The Discriminatory practices column lists examples of discriminatory behaviors, such as stereotyping, misgendering, and invalidation of experiences of racism. The table highlights the alignment of these measures with identified discriminatory practices.

Navigate back to Table 2.

## Table 4 Long description

The table presents structural-level measures for intersectional healthcare and their alignment with discriminatory practices. It includes three columns: Measure, Description, and Discriminatory practices. The Measure column lists measures such as Inclusive and discrimination-critical therapy materials, Discrimination-critical therapy concepts, Address barriers to medical school admissions, and Address barriers to healthcare access. The Description column provides details on each measure, including developing assessment tools, therapy concepts, and addressing barriers. The Discriminatory practices column highlights issues like limited awareness of discrimination-sensitive tools, absence of racism-sensitive therapy concepts, and mental healthcare institutions as exclusive spaces. The table has multiple rows, each detailing specific measures and their descriptions alongside corresponding discriminatory practices.

Navigate back to Table 4.

## Table 3 Long description

The table presents organizational-level measures for intersectional healthcare and their alignment with discriminatory practices. It has five rows and two columns. The first column lists measures such as providing information materials in multiple languages, offering interpretation services, considering religious needs, introducing appointment scheduling systems, implementing group therapy, involving leadership in anti-discrimination, investing resources in anti-discrimination infrastructure, adopting diversity-oriented hiring practices, regularly discussing sensitive topics, establishing mandatory workshops, and creating standard operating procedures. The second column describes discriminatory practices such as structural language barriers, cis-normative infrastructures, hostile institutional climate, lack of space for marginalized experiences, absence of safe infrastructure, lack of understanding in the work environment, inadequate critical reflection, limited provider skills, and absence of guidelines on gender diversity. The table highlights the need for inclusive practices to address and mitigate discriminatory practices in healthcare settings.

Navigate back to Table 3.
