# Supplementary material for: Measures for intersectional mental healthcare: results from a participatory interview and focus group study with psychosocial counsellors, people with experience of using mental health services and providers
Source: BJPsych Open. 2026 Aug 10;12(5):e210. doi: 10.1192/bjo.2026.12026 (PMC13458194; doi:10.1192/bjo.2026.12026)
Supplement: Supplementary file 1 [file S2056472426120262sup001.docx]

**Online Supplement**

**Measures for intersectional mental healthcare. Results from a participatory interview and focus group study with psychosocial counsellors, mental healthcare service users and providers**

A) **Focus group guide**

The interview guide was translated from German to English.

| **Guiding questions** | **Further questions for clarification** |
| --- | --- |
| **Reflexion Questions** (with reference to Phase 1)   1. We have just presented the main findings of the study. To what extent do these findings align with your own experiences? Where do you see differences between our study results and your own experiences? 2. From your perspective, how understandable and plausible are the findings? 3. Do you see alternative ways of interpreting the results? If so, what are they? 4. Based on your own experience, what connections do you see between discrimination related to mental illness and other systems of discrimination such as racism, sexism, classism, or ableism? | What surprises you?  What did you not expect in this way?  Do the findings trigger any emotional reactions?  Are there alternative explanatory models or approaches?  Do you have any objections to the proposed interpretations? |
| **Evaluation Questions I** (with reference to Phase 1)   5. As described, intersectional discrimination means not looking at different forms of discrimination separately, but in relation to one another. How do you evaluate the concept of intersectionality for our study? 6. How would you assess the overall design of the study? Are there aspects we should have approached differently? If so, which ones? | What strengths and weaknesses of the study are mentioned? |
| **Evaluation Questions II** (Phase II)   7. Looking ahead: In your view, what could anti-discriminatory mental healthcare look like? 8. Based on this, what practical implications would you draw from the study findings? What measures would you consider helpful? | What measures are mentioned at the individual, institutional, and structural levels?  Do participants see a need for action? If so, for whom?  Which measures are assessed helpful and which feasible?  Are specific measures for the Bochum site mentioned?  How is the potential for change assessed? |
| **Closing**   10. Is there anything we have not addressed that you would like to add? 11. How was the focus group discussion for you? |  |

B) **Researcher reflexivity statement**

Our research team engaged in regular reflexive meetings throughout the research process to interrogate how our own positions, assumptions, and experiences shaped the study. These meetings provided a structured space to surface implicit background assumptions about the topic, challenge what we tended to take for granted, and consider how our social positioning within academic and societal power structures might influence the questions we asked, the interactions we had with participants, and the interpretations we produced.

In practical terms, our sessions often began from concrete moments in the field or during analysis. For example, when several team members initially read participants’ accounts through a predominantly individual‑clinical lens, our discussions made visible how our professional training predisposed us to privilege intrapsychic explanations over structural ones. By naming this bias explicitly, we were able to revisit our coding and develop categories that gave greater weight to structural violence, genderism, racism, or organisational conditions that participants themselves emphasised. In another instance, a team member who shared key identity markers with a subgroup of participants noticed that she was more inclined to interpret their narratives as “resistant” rather than “non‑compliant.” Bringing this observation into the reflexive space helped the team recognise how identification can open up certain readings while closing down others, and led us to systematically check where empathy might be amplifying or muting particular themes.

The meetings also foregrounded power and positionality within the research relationship. As mostly university‑affiliated researchers with secure employment, we recognised that our institutional status and relative social privilege could make participants more cautious in how they spoke about organisations, colleagues, or experiences of discrimination. Discussing these dynamics encouraged us to adapt our interviewing style, to make power imbalances explicit where appropriate, and to interpret silences, hesitations, or guarded statements not simply as “lack of data” but as meaningful responses to the research context. Similarly, reflecting on differences in gender, sexual orientation, racialisation, migration history, class, and professional role within the team highlighted how certain topics felt more “available” or “speakable” to some researchers than to others. Building these insights into our analytic conversations meant that we could actively invite marginalised perspectives within the team and avoid letting the most institutionally powerful voices set the interpretive agenda.

Methodologically, these reflexive sessions enhanced the quality and trustworthiness of our research in several ways. First, they increased transparency by making our limitations of perspective, our assumptions, investments and areas of unawareness explicit rather than implicit. Second, they strengthened the credibility and depth of our interpretations: revisiting coding decisions in light of reflexive discussions often led to more nuanced and multi‑layered categories, including contradictions and ambivalence that we had initially smoothed over. Third, they contributed to ethical rigour, as thinking carefully about how our positionalities shaped recruitment, interviewing, and analysis allowed us to better account for the potential harms of misrepresentation, silencing, or over‑pathologising particular groups. Overall, incorporating collective reflexivity as a systematic element of the research design supported a more accountable, context‑sensitive and critically engaged knowledge production.
